# *Rickettsia slovaca* and *R. raoultii* in Tick*-*borne Rickettsioses

**DOI:** 10.3201/eid1507.081449

**Published:** 2009-07

**Authors:** Philippe Parola, Clarisse Rovery, Jean Marc Rolain, Philippe Brouqui, Bernard Davoust, Didier Raoult

**Affiliations:** Unité des Rickettsies, Marseille, France (P. Parola, C. Rovery, J.M. Rolain, P. Brouqui, D. Raoult); Direction Régionale du Service de Santé des Armées, Toulon, France (B. Davoust)

**Keywords:** DEBONEL, TIBOLA, Rickettsia slovaca, Rickettsia raoultii, *RpA4*, Dermacentor, ticks, dispatch, Rickettsia

## Abstract

Tick-borne lymphadenopathy (TIBOLA), also called *Dermacentor*-borne necrosis erythema and lymphadenopathy (DEBONEL), is defined as the association of a tick bite, an inoculation eschar on the scalp, and cervical adenopathies. We identified the etiologic agent for 65% of 86 patients with TIBOLA/DEBONEL as either *Rickettsia slovaca* (49/86, 57%) or *R. raoultii* (7/86, 8%).

In 1968, *Rickettsia slovaca,* a spotted fever group (SFG) rickettsia, was isolated from *Dermacentor marginatus* ticks in the former Czechoslovakia before being detected in *D. marginatus* or *D. reticulatus* ticks throughout Europe (Figure 1) (*1*). In 1997, *R. slovaca* was described as a human pathogen and an agent of tick-borne lymphadenopathy (TIBOLA) (*2*). This syndrome, also called *Dermacentor*-borne necrosis erythema and lymphadenopathy (DEBONEL), is defined as the association of a tick bite, an inoculation eschar on the scalp, and cervical lymphadenopathies (*3*).

**Figure 1:**
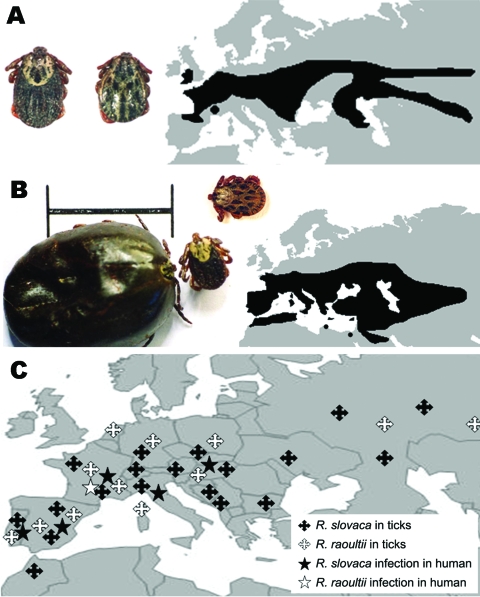
*Dermacentor reticulatus*, the ornate dog tick (A) (female, left; male, right), and *D. marginatus*, the ornate sheep tick (B) (engorged female, left; unfed female, center; male, right; scale bar = 1 cm), and their distribution. *D. marginatus* is most frequently found in Mediterranean areas of Europe with dense bush and tree cover and is common under oak and pine vegetation. It also has a restricted distribution in North Africa, in the cooler and more humid areas associated with the Atlas Mountains. Adults infest large mammals such as sheep, cattle, goats, and wild boars. Larvae and nymphs feed mostly on small mammals and medium sized carnivores. *D. reticulatus* is most frequently found in colder northern areas of western Europe and the former Soviet Union, with high humidity and mild winters. *D. reticulatus* is primarily a tick of dogs and carnivores, but it can be found on ungulates such as sheep, cattle, and horses (*9*). *D. marginatus* and *D. reticulatus* have been suggested as reservoirs of *R. slovaca* and *R. raoultii,* which are maintained in ticks through transstadial and transovarial transmission. Therefore, the geographic distribution of these rickettsiae likely parallels that of *Dermacentor* ticks (C).

Since 1999, several rickettsial genotypes, called DnS14, DnS28, and RpA4, have been detected in *Dermacentor* spp. ticks throughout Europe (Figure 1). Isolates have been obtained and shown to belong to a unique new SFG rickettisia species named *R. raoultii* (*4*). In 2002, *R. raoultii* DNA was detected in a *D. marginatus* tick taken from the scalp of a patient in whom TIBOLA/DEBONEL developed in France (*4*). Moreover, DNA of what is now known to be *R. raoultii* has been found in the blood of 1 patient with TIBOLA/DEBONEL (*5*). The goal of this study was to identify the rickettsial agents in patients with TIBOLA/DEBONEL symptoms and in those who had an isolated tick bite on the scalp.

## The Study

**Figure 2:**
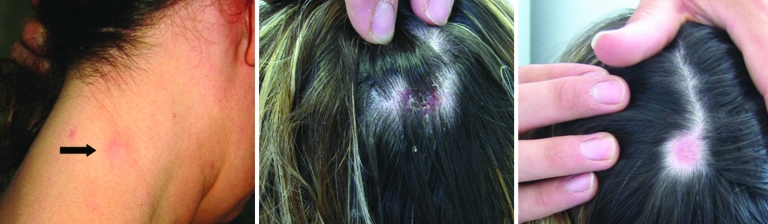
Typical signs of TIBOLA (tick-borne lymphadenopathy)/DEBONEL (*Dermacentor*-borne necrosis erythema and lymphadenophy). Here, infections were caused by *Rickettsia slovaca* , resulting in cervical lymphadenopathy (left panel, arrow), inoculation on the scalp (middle panel), and residual alopecia 4 weeks later (right panel).

We included all patients with TIBOLA/DEBONEL symptoms (Figure 2) and those who had an isolated tick bite on the scalp without any symptoms from whom samples (serum, skin biopsy, or ticks harvested from the scalp) were received at our laboratory from January 2002 through December 2007. Epidemiologic and clinical data were collected retrospectively. The study was approved by the ethics committee of the Medicine School of Marseille under reference 08-008.

Immunoglobulin (Ig) G and IgM titers against rickettsial antigens were estimated by microimmunofluorescent assay; results were verified by Western blot and cross-absorption studies (*3*). Ticks found on persons and skin biopsy specimens were cultured on human embryonic lung cells (*6*). These samples were also used to amplify and identify outer membrane protein A–encoding gene fragments of rickettsiae by PCR (*3*). Also, the so-called suicide PCR-assay was used with acute-phase serum samples (*1*).

Among 98 study patients, 86 were classified as TIBOLA/DEBONEL patients. Twelve (12.2%) patients made up the second group with an isolated tick bite. All but 1 patient, who was bitten in Belgium, were bitten in France. Tick bites more frequently occurred from February through May (50/86, 58.1%). Because of results of serologic techniques, we could conclude that 66 (84.6%) of 78 TIBOLA/DEBONEL patients with obtained serum specimens had a recent rickettsial disease. Western blot and cross-adsorption analyses enabled detection of antibodies specifically directed against *R. slovaca* and *R. raoultii* in 34 and 4 patients, respectively (Appendix Table).

Two patients who were infected with *R. slovaca* were found to be co-infected with *Coxiella burnetii* in an acute form of Q fever. Serologic testing was performed in 12 patients with isolated tick bites, and results were negative in all cases. A total of 19 skin biopsy specimens were obtained. Four *R. slovaca* infections were diagnosed by regular PCR, and 3 isolates of this rickettsia were obtained. The suicide PCR on acute-phase serum samples identified 1 additional case of *R. slovaca* infection. Because of molecular tools and culture, 6 patients received the diagnosis of an *R. slovaca* infection, including 5 who did not receive a diagnosis by serologic assays (Appendix Table). Ticks removed from 28 TIBOLA/DEBONEL patients consisted of 23 *D. marginatus* (88.4%), 2 *Dermacentor* spp., 1 *Haemaphysalis punctata*, and 2 that were not identified. Overall, the tick studies enabled us to suggest the diagnosis of *R. slovaca* and *R. raoultii* infections in 10 and 3 patients, respectively, whose conditions were not diagnosed with previous tests (Appendix Table).

All DNA sequences obtained showed 100% identity with *R. raoultii* or *R. slovaca*, excluding the coexistence of several rickettsiae in the corresponding samples. According to our investigations, 49 (57%) of 86 patients with TIBOLA/DEBONEL had probable or certain *R. slovaca* infections, and 7 (8%) of 86 had probable *R. raoultii* infections. The characteristics of these patients are shown in the Table.

**Table:** Characteristics of TIBOLA/DEBONEL patients with certain or probable *Rickettsia slovaca* infection compared with patients with certain or probable *R. raoultii* infection*

Characteristic	TIBOLA/DEBONEL patients, n = 86		
	No*. R. slovaca* infections (%), n = 49†‡	No*. R. raoultii* infections (%), n = 7†	p value
Female sex	33/49 (67)	7/7 (100)	0.04
Mean age, y	32	32	0.90
Age <12 y	20/49 (41)	3/7 (43)	0.46
Hiking or recreational activities such as a walk in the forest	21/28 (75)	4/5 (80)	0.44
Fever§	21/39 (54)	4/5 (80)	0.27
Painful eschar	14/22 (64)	3/3 (100)	0.30
Painful adenopathies	18/26 (69)	5/5 (100)	0.20
Face edema	6/31 (19)	2/5 (40)	0.30
Rash	7/30 (23)	1/5 (20)	0.68
Headache	16/30 (53)	4/4 (100)	0.10
Alopecia	16/27 (59)	0/4	0.09
Asthenia	23/33 (70)	5/5 (100)	0.20
Prolonged asthenia¶	10/29 (35)	2/4 (50)	0.46
Chronic asthenia#	4/28 (14)	1/4 (25)	0.51

*Certain cases were those with positive culture, PCR, or suicide PCR results in blood or skin biopsy samples or with lymph node aspirates. Probable cases were those with identification by PCR and sequencing of the corresponding *Rickettsia* spp. in ticks, Western blot results demonstrating *R. slovaca*– or *R. raoultii*–specific antibodies, or a cross-absorption assay demonstrating specific antibodies against *R. slovaca* or *R. raoultii*. TIBOLA, tick-borne lymphadenopathy; DEBONEL, *Dermacentor*-borne necrosis erythema and lymphadenopathy. †Denominators indicate the number of patients for whom the criterion was available. ‡Includes 14 patients previously reported (*7*). §Temperature >37°5C. ¶Self-reported, persistent asthenia of 1 to 6 months. #Self-reported persistent or relapsing asthenia of >6 consecutive months.

## Conclusions

We report 86 patients with TIBOLA/DEBONEL; this group includes 14 patients whose conditions had been preliminarily reported (*7*). We also describe several cases caused by the emerging pathogen *R. raoultii* (*4*), including patients with indirect molecular evidence of infection because the pathogen was detected in the ticks that had bitten them. Original findings also include facial edema as a new clinical feature in TIBOLA/DEBONEL, and the report of the second patient co-infected with *R. slovaca* and *C. burnetii* (*8*). Because acute Q fever, a worldwide zoonosis, may be asymptomatic, we recommend that patients infected with tick-borne pathogens also undergo testing for concurrent infections with *C. burnetii*.

No TIBOLA/DEBONEL cases were recorded during the warmest summer months; peak incidence occurred during March–May and during September–November, linked with the activity of *Dermacentor* ticks in Europe (Figure 1) (*9*). However, to date, we have no explanation for the finding that children and women are at higher risk for TIBOLA/DEBONEL or why *D. marginatus* and *D. reticulatus* ticks prefer to bite persons on the scalp. A possible explanation could be that *Dermacentor* ticks usually bite hairy domestic and wild animals and the longer hair of women and children may attract them.

One of the most remarkable findings of this work is the proportional importance of *R. slovaca* in TIBOLA/DEBONEL patients, compared with *R. raoultii*. In 2006, Ibarra et al. reported on 14 persons in Spain who had a *D*. *marginatus* tick attached to the scalp (*10*). All ticks were found to be infected by rickettsiae: 8 (58%) were infected by *R. slovaca,* and 6 (42%) by *R. raoultii*. In 10 of the patients, TIBOLA/DEBONEL symptoms developed, including in all 8 of the patients who had been bitten by a tick infected by *R. slovaca* and in 2 of the 6 patients who had been bitten by a tick infected by *R. raoultii*. *R. slovaca* was more significantly associated with TIBOLA/DEBONEL patients than was *R. raoultii* (p<0.05) (*10*). Here, focusing on the studies of ticks removed from TIBOLA/DEBONEL patients, we found that 12 of 19 ticks harbored *R. slovaca*, whereas only 3 of 19 harbored *R. raoultii* (p = 0.047). In the patients with asymptomatic tick bites, from whom 9 ticks were obtained, all ticks positive by PCR harbored *R. raoultii*.

Moreover, *R. raoultii* seems to be more highly prevalent in *D. marginatus* and *D. reticulatus* ticks in nature than is *R. slovaca*. Although comparing field surveys of ticks is difficult because of the sampling methods, the sizes of the samples, and the potential PCR inhibitors, *R. raoultii* has been more frequently detected in *D. marginatus* ticks than has *R. slovaca*. In southeastern Spain, 73% of 101 *D. marginatus* ticks were infected by *R. raoultii* and 27% by *R. slovaca* (*11*). Similar differences have been shown in Germany, Portugal, the Netherlands, and Spain (*12**–**15*). Although interpreting these data definitively is difficult, the recurrence of similar published results by different teams suggests that exposure to *R. raoultii* through the bite of a *Dermacentor* spp. tick is likely more frequent than exposure to *R. slovaca*. However, more cases of *R. slovaca* infection have been recorded, which suggests that *R. raoultii* is less pathogenic.

TIBOLA/DEBONEL is a newly recognized disease, and its incidence is likely underestimated. In our laboratory, TIBOLA/DEBONEL is the most frequently reported rickettsial disease, except during the dry summer period. Doxycycline remains the treatment of choice, with new macrolides as alternative treatments (*1*). Although we report 6 more cases of *R. raoultii* infection in addition to the 2 recently reported (*4**,**5*), this *Dermacentor*-borne rickettsia seems to be less pathogenic than *R. slovaca*.

## Supplementary Material

Appendix Table Results of microbiologic investigations regarding rickettsial diseases of patients with TIBOLA/DEBONEL or tick-bite*
