# Supplementary material for: Rickettsia slovaca and R. raoultii in Tick-borne Rickettsioses
Source: Emerg Infect Dis. 2009 Jul;15(7):1105–8. doi: 10.3201/eid1507.081449 (PMC2744242; doi:10.3201/eid1507.081449)
Supplement: Appendix Table — Results of microbiologic investigations regarding rickettsial diseases of patients with TIBOLA/DEBONEL or tick-bite* [file 08-1449_appT-s1.pdf]

Appendix Table. Results of microbiologic investigations regarding rickettsial diseases of patients with TIBOLA/DEBONEL or tick-bite*

Patients	Serologic assays (MIF assay and WB)†				Suicide PCR‡ of acute-phase serum sample				Standard PCR and/or culture of cutaneous biopsy specimens				Tick study				All methods		
	No. pos/ no. tested	Rs	Rr	ND	No. tested	Rs	Rr	Neg	No. tested	Rs	Rr	Neg	No. tested	Rs	Rr	Neg	Rs	Rr	ND
With TIBOLA/ DEBONEL (n = 86)	66/78	34	4	28	36	1§	0	35	19	5¶	0	13	19	12#	3	4	49**,††	7**	30
Had isolated tick bite (n = 12)	0/5	–	–	–	5	0	0	0	–	–	–	–	9	0	5	4	0‡‡	5‡‡	7

*TIBOLA, tick-borne lymphadenopathy; DEBONEL, *Dermacentor*-borne necrosis erythema and lymphadenophy; MIF, microimmunofluorescent; WB, Western blot; pos, positive; Rs, *Rickettsia slovaca* specific diagnosis or identification; Rr, *Rickettsia raoultii* specific diagnosis or identification; ND, specific rickettsia involved was not determined; neg, negative.

†MIF assay using 7 spotted fever group rickettsial antigens (*R. conorii*, *R. aeschlimannii*, *R. massiliae*, *R. helvetica, R. slovaca, R. raoultii, R. felis*), a typhus group antigen, *R. typhi*, and *Coxiella burnetii*. For rickettsial antigens, titers ≥64 for immunoglobulin (Ig) G and ≥32 for IgM, as well as seroconversion or a 4-fold increase in antibody titers from early to late serum specimens, were considered MIF positive. When cross-reactions were noted among several rickettsial antigens, a rickettsial antigen was considered to indicate presence of the infectious agent if titers of IgG and/or IgM antibody against this antigen were at least 2-fold higher than titers of IgG and/or IgM antibody against other rickettsial antigens. When titers differed by ± 1 standard dilution, WB assays and cross absorption studies were performed.

‡Nested PCR in which both the template DNA fragment and the primers are used only once. (Primers available on request.) All positive PCR amplicons were sequenced with the primers used for PCR, for precise identification of the infecting *Rickettsia* species. As negative controls, introduced for every 5 patient specimens, we used cardiac valve biopsy specimens obtained from patients who had undergone a valvular replacement for a degenerative disease. No positive controls were used.

§Sample from patient negative by serologic assays.

¶Includes 4 samples from patients negative by serologic assays.

#Includes 10 samples from patients negative by serologic assays, culture, and molecular methods.

**p<0.05 (by Mantel-Haenszel test) between these 2 groups.

††Including 2 cases with coinfection with *C. burnetii*, the agent of Q fever. The first case occurred in the patient described above with neurologic symptoms (diagnosed on the basis of an anti–phase II IgG titer of 100 and an anti–phase II IgM titer of 50). The second case-patient was a pregnant woman with no symptoms other than the TIBOLA/DEBONEL (diagnosed on the basis of an anti–phase II IgG titer of 400 and an anti–phase II IgM titer of 100).

‡‡p<0.05 (by Mantel-Haenszel test) between these 2 groups.
